# Conjunctive Processing of Locomotor Signals by the Ventral Tegmental Area Neuronal Population

**DOI:** 10.1371/journal.pone.0016528

**Published:** 2011-01-27

**Authors:** Dong V. Wang, Joe Z. Tsien

**Affiliations:** 1 Key Laboratory of MOE and STCSM, Shanghai Institute of Brain Functional Genomics, East China Normal University, Shanghai, China; 2 Brain and Behavior Discovery Institute, Medical College of Georgia, Georgia Health Sciences University, Augusta, Georgia, United States of America; Université Pierre et Marie Curie, France

## Abstract

The ventral tegmental area (VTA) plays an essential role in reward and motivation. How the dopamine (DA) and non-DA neurons in the VTA engage in motivation-based locomotor behaviors is not well understood. We recorded activity of putative DA and non-DA neurons simultaneously in the VTA of awake mice engaged in motivated voluntary movements such as wheel running. Our results revealed that VTA non-DA neurons exhibited significant rhythmic activity that was correlated with the animal's running rhythms. Activity of putative DA neurons also correlated with the movement behavior, but to a lesser degree. More importantly, putative DA neurons exhibited significant burst activation at both onset and offset of voluntary movements. These findings suggest that VTA DA and non-DA neurons conjunctively process locomotor-related motivational signals that are associated with movement initiation, maintenance and termination.

## Introduction

The ventral tegmental area (VTA) is widely believed to play an essential role in reward, motivation and drug addiction [1]–[5]. Electrophysiological properties of putative dopamine (DA) neurons in the VTA have been extensively studied in previous studies, showing that unexpected reward (e.g., food, juice) and reward cues (conditioned stimuli) evoke a brief burst activity of the DA neurons [6]–[9]. These DA neurons' responsiveness appears to encode a wide range of novel and reward-related events through a prediction error rule [6]–[8]. When it is fully predicted, DA neurons show very limited or no responses to a food or juice reward [6]–[8]. Together with many other studies involving lesions and genetic manipulations, it is suggested that dopamine activity is more likely to be associated with motivation and incentive “wanting” rather than the hedonic impact of reward [3], [10], [11]. While non-DA neurons are presumed to play an important role in regulation of DA neurons in the VTA, few studies have examined their roles in animal's motivational behaviors.

In the ventral tegmental area (VTA), overall 33–45% of the neurons are estimated to be non-DA neurons in rodents [12]–[14]. Of note, distributions of the non-DA neurons range from around 10–60% in different subregions of the VTA [13], [14], suggesting a great heterogeneity in the VTA. The majority of these non-DA neurons are GABAergic neurons, which are presumed to play an important role in local inhibition of DA neurons in the VTA [14], [15]. Importantly, VTA GABAergic neurons are also involved in regulation of striatal and cortical neurons through their projecting axons, e.g., an estimated 58% of mesoprefrontal and 20% of mesoaccumbal projection neurons are GABAergic [16]–[19]. The anatomic complexity and heterogeneity of the VTA non-DA neuron may explain its diverse functions that are possibly involved in reward, psychomotor control and addiction behaviors [20]–[23].

It has been reported that VTA non-DA neurons increase their firing rates significantly during active wakefulness and rapid eye movement (REM) sleep, relative to quiet wakefulness [21], [23]. In particular, VTA non-DA neuron firing rates increase markedly at the initiation of movement [21] and their activity correlates with the velocity or acceleration of animal's movement [24]. These results suggest that VTA non-DA neurons play an important role in animal's locomotor activity. However, the role of the VTA non-DA neurons in sustained locomotor activity has not been quantitatively well examined [21]. More importantly, whether the DA neurons in the VTA may play a role in motivation-based locomotor activity is still not clear. Wheel running is a widely reported behavior performed by rodents and is believed to be possibly rewarding or motivation-driven [25]–[27]. Therefore, wheel running is a potential good model for studying motivation and locomotor activity. To further understand the role of VTA DA and Non-DA neurons in animal's motivational locomotor behaviors, we subjected mice to voluntary wheel running while we simultaneously recorded multiple neuronal activities in the VTA area. Our results suggest that VTA putative DA and non-DA neurons conjunctively process locomotor-related motivational signals that are associated with movement initiation, maintenance and termination.

## Results

### Muti-tetrode recording in the VTA of freely behaving mice

**Figure 1:**
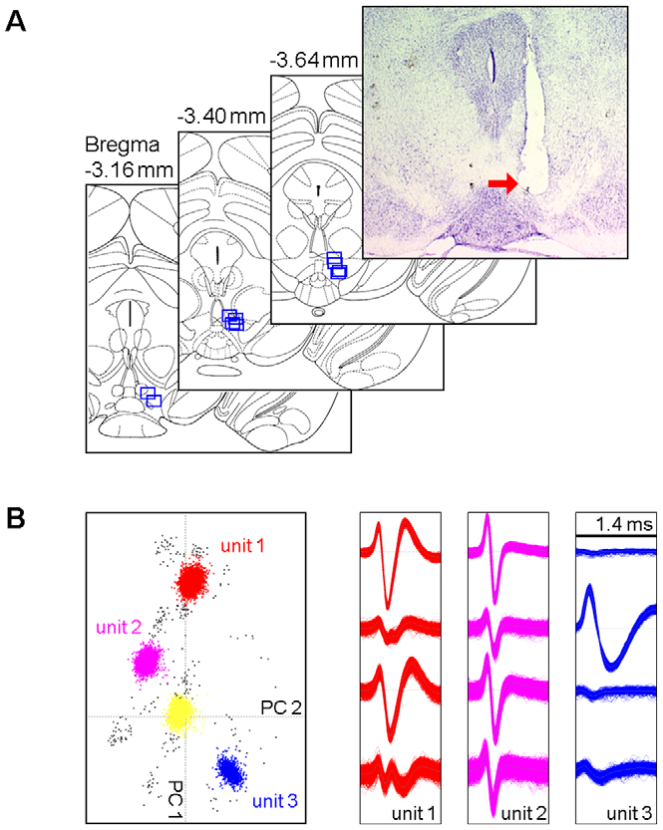
Muti-tetrode recording and spike sorting. (A) Electrode array track shown on an example coronal brain section (top-right) and locations of the electrode array tip from 10 mice on the atlas section diagrams [44]. (B) Left panel, an example of spike sorting using principle component analysis (Plexon OfflineSorter). Here, multiple units were recorded simultaneously from one tetrode; red/purple/blue dots represent isolated units 1–3, respectively; yellow dots represent un-isolated spikes and noises; black dots represent overlapping spike waveforms, which would be manually assigned to units 1–3. Middle and right panels, representative spike waveforms for the units 1–3. Note that only unit 3 was classified as putative DA neuron, while units 1 and 2 were classified as non-DA neurons.

We implanted movable bundles of 8 tetrodes (32 channels) into the VTA of the right hemisphere. Data from a total of 10 mice from which we recorded both putative DA and non-DA neurons were used for analyses, and the recording electrodes' positions were confirmed by histology (Figure 1A). We only included units with clear spike waveforms and isolations for further analyses (for examples of well-isolated units recorded from one tetrode, see Figure 1B_units 1–3). Overall, a total of 72 well-isolated units were recorded in the VTA area. Of these, 25 were classified as putative DA neurons according to previously established criteria [28]–[30] (see Materials and Methods), and the other 47 units were classified as non-DA neurons. These putative DA neurons typically exhibited broad, tri-phasic spike waveforms (see Figure 1B_unit 3 for an example). They all showed low baseline firing rates (0.5–10 Hz) and regular firing pattern. On the other hand, the vast majority of the classified non-DA neurons (41/47) showed high baseline firing rates (>10 Hz) and movement modulated firing changes [21], [23].

**Figure 2:**
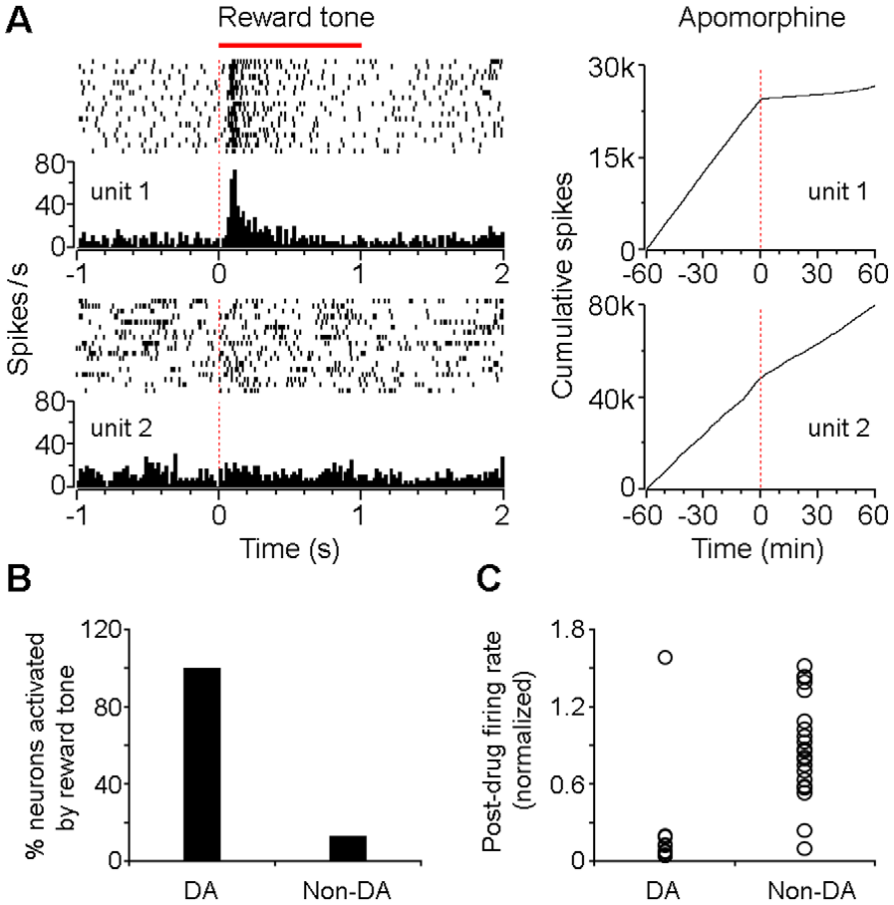
Classification of VTA putative DA and non-DA neurons. (A) Left panels, peri-event rasters and histograms of two simultaneously recorded VTA neurons (unit 1: putative DA neuron; units 2: non-DA neuron) in response to the conditioned tone (5 kHz, 1 sec) that reliably predicted food delivery. Right panels, cumulative spike activity of the same two neurons before and after (-60-0 and 0–60 min, respectively) the injection of the dopamine receptor agonist apomorphine (1 mg/kg, i.p.). (B) Percentages of classified putative DA (100%; n  =  25) and non-DA neurons (13%; 6/47) that were significantly activated by the conditioned tone that reliably predicted food delivery. (C) Normalized firing rates of putative DA and non-DA neurons after the injection of apomorphine (firing rates averaged for 30 min). Note that 9 out 10 putative DA neurons tested showed significant suppression (≤ 20% baseline firing rates), while the majority of the non-DA neurons (20/22) showed limited or no change of firing rate by apomorphine (1 mg/kg, i.p.).

Moreover, these classified putative DA neurons exhibited significant activation after onset of the conditioned tone that reliably predicted subsequent food delivery, while VTA non-DA neurons typically showed no response to the conditioned tone (for examples of two simultaneously recorded putative DA and non-DA neurons, see Figure 2A_left panels). Overall, all the classified putative DA neurons (n = 25) showed significant activation after onset of the conditioned tone that predicted subsequent food delivery (*P*<0.01, Wilcoxon signed-rank test), while very few VTA non-DA neurons (13%; 6/47) showed significant activation in response to the conditioned tone (Figure 2B).

We also injected the mice with the dopamine receptor agonist apomorphine (1 mg/kg, i.p.) which has been mainly shown to inhibit the activity of the DA neuron [28], [30]. As shown in the cumulative spike activity histograms, the putative DA neuron (unit 1) exhibited strong suppression, while the non-DA neuron (unit 2) showed no significant firing changes after the injection of apomorphine (Figure 2A, right panels). We randomly tested 32 recorded VTA neurons (including 10 putative DA and 22 non-DA neurons) and our pharmacology results confirmed that 9 out of 10 putative DA neurons showed significant suppression ( = 20% baseline firing rate), while the majority of VTA non-DA neurons (20/22) showed limited or no change in firing rate after the injection of apomorphine (Figure 2C).

### Rhythmic activity of the VTA non-DA neuron during wheel running

**Figure 3:**
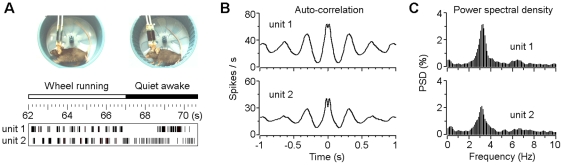
Rhythmic activity of VTA non-DA neurons during wheel running. (A) Rasters of two simultaneously recorded VTA non-DA neurons during voluntary wheel running and quiet wakefulness. (B and C) Auto-correlation and power spectral density (PSD) analyses suggest that the same two neurons (as shown in A) showed strong rhythmic activity (at a cycle of 0.31 sec on average) during wheel running.

To examine the correlation between the VTA neuron activity and animal's motivational locomotor behaviors, we subjected mice to voluntary wheel running while simultaneously recording neuronal activities in the VTA. We found that VTA non-DA neurons showed significant change of firing rates and/or firing patterns during wheel running compared with quiet wakefulness (Figure 3A). And more importantly, these non-DA neurons exhibited significant rhythmic activity during wheel running. As an example, both auto-correlation analyses (Figure 3B) and power spectral density analyses (Figure 3C) indicate that the two VTA non-DA neurons exhibited strong rhythmic activity (at a cycle of 0.31 sec on average) that correlated well with the animal's wheel running cycle (0.31±0.06 sec; mean ± s.d.; calculated by limb-movement cycles).

**Figure 4:**
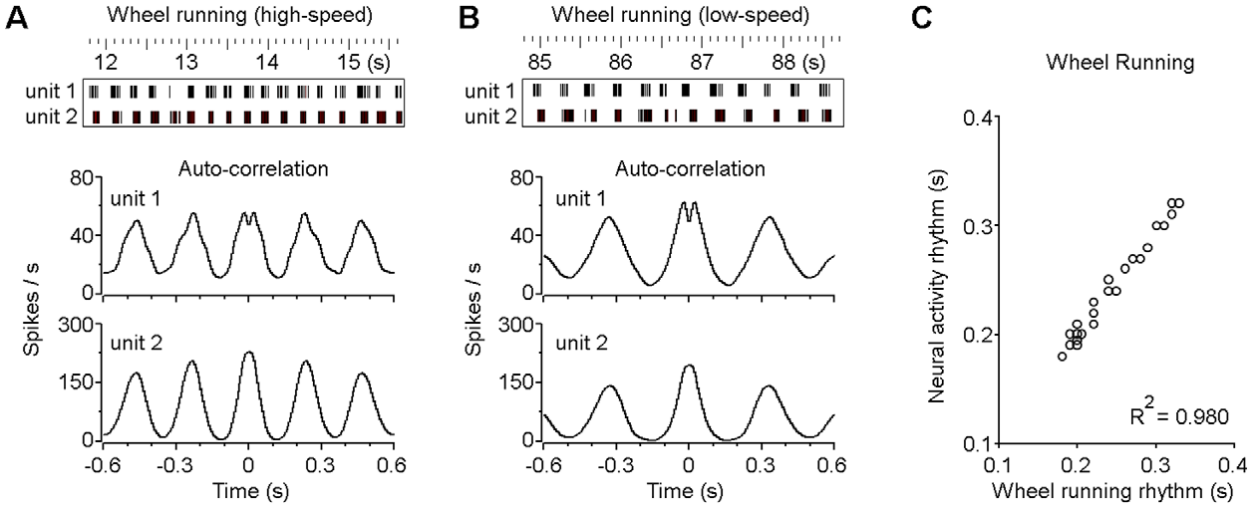
Activity of the VTA non-DA neuron correlates with the wheel running rhythm. (A and B) Rasters (upper panels) and smoothed auto-correlation histograms (lower panels) of two simultaneously recorded VTA non-DA neurons during high-speed (A) and low-speed (B) voluntary wheel running. (C) VTA neuron activity correlates with the limb-movement rhythm during wheel running.

We also compared the neuronal activities between high-speed and low-speed voluntary wheel running. Our auto-correlation analyses revealed that the activity of the VTA non-DA neurons correlated well with the wheel running speed (Figure 4A and B). Population analysis revealed that the activity rhythms of the VTA non-DA neurons correlated tightly with the wheel running rhythms (Figure 4C). These results suggest that VTA non-DA neurons play an important role in voluntary locomotor activity that is associated with sustained rhythmic running behaviors.

**Figure 5:**
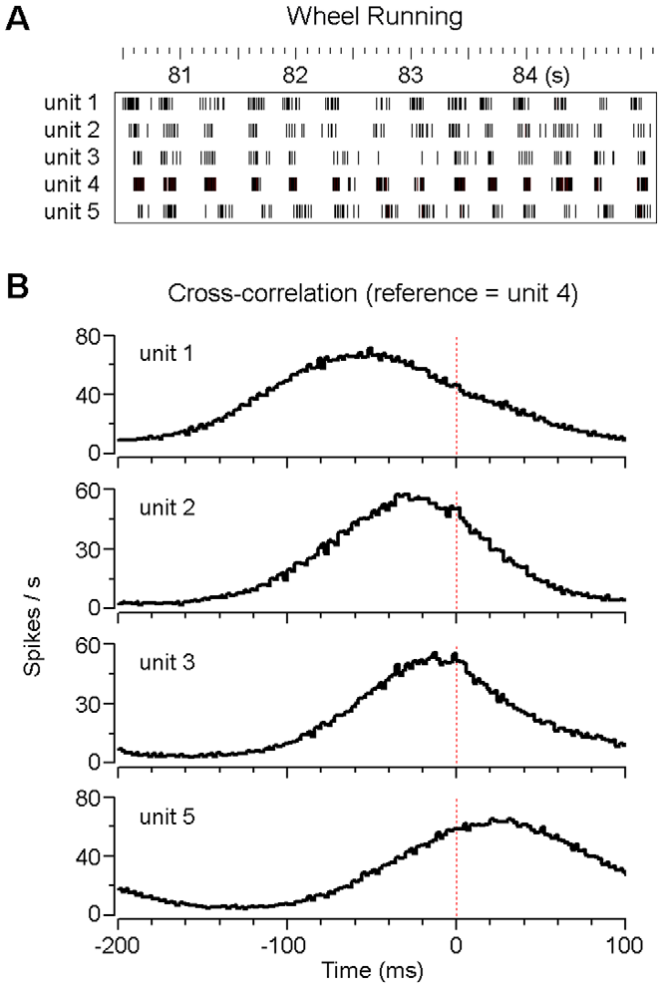
Phase-specific firing of VTA non-DA neurons during wheel running. (A) Rasters of five simultaneously recorded VTA non-DA neurons during voluntary wheel running. (B) Cross-correlation histograms show that the same five neurons (as shown in A) fire preferentially at specific phases of each cycle and in sequence during voluntary wheel running (unit 4 was used as the reference for cross-correlation calculation).

We next asked whether the VTA non-DA neuron fired at a specific phase of each wheel running cycle. Our analyses suggested that individual VTA non-DA neurons fired preferentially at specific phases of the wheel running cycle. As shown in Figure 5A, five VTA non-DA neurons (units 1–5) were recorded simultaneously during wheel running. These non-DA neurons showed strong rhythmic activity, and more importantly, phase-specific and phase-locked firing in each wheel running cycle. By calculation of their cross-correlations (we chose the most rhythmic unit 4 as the reference), our results suggested that units 1–5 fired sequentially at specific phases of the wheel running cycle (Figure 5B).

### Rhythmic activity of the VTA putative DA neuron during wheel running

Dopamine has been suggested to play an important role in locomotor activity and motor learning [31]–[34]. Dopamine transmission in the nucleus accumbens (which mainly receives dopaminergic input from the VTA) is important for locomotor activity such as sensitization induced by psychostimulants, opioids and stressors [32]. We thus set out to examine whether and how the activity of the VTA putative DA neurons was involved in motivated locomotor behaviors such as wheel running. To address this question, data from simultaneously recorded putative DA and non-DA neuron pairs were used for analyses.

**Figure 6:**
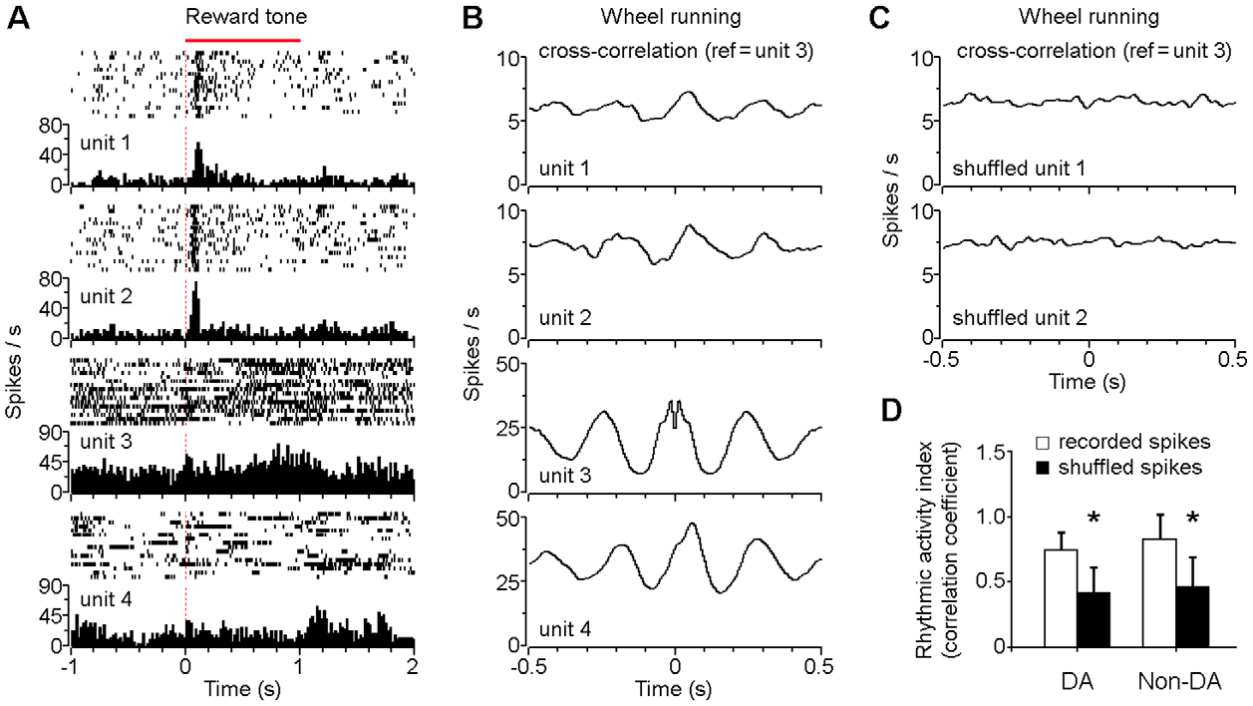
Rhythmic activity of both putative DA and non-DA neurons during wheel running. (A) Peri-event rasters and histograms of four simultaneously recorded VTA neurons in response to the conditioned tone that reliably predicted food delivery (units 1 & 2: putative DA neurons; units 3 & 4: non-DA neurons). (B) Smoothed cross-correlation histograms of the same four neurons (as shown in A) during voluntary wheel running. Unit 3 was used as the reference for cross-correlation calculations. (C) Smoothed cross-correlation histograms of shuffled units 1 and 2 (randomized spikes). The same unit 3 (as shown in B) was used as the reference for cross-correlation calculations. (D) Correlation coefficient analyses and comparisons between the recoded and shuffled spikes. A set of simulated sine oscillation curves y  =  sin (ax + b) were used as the reference for the correlation coefficient analysis (see Materials and Methods). n = 14 and 29 for DA and non-DA neurons, respectively; *P<0.001, Student's paired *t*-test. Error bars represent s.d.

As shown in Figure 6A, four neurons were simultaneously recorded in the VTA. The two putative DA neurons (units 1 and 2) increased their activity significantly after onset of the conditioned tone that predicted subsequent food delivery, consistent with previous studies showing that reward and reward cues caused a short-latency (50–110 ms) and short-duration (∼200 ms) burst activity of the DA neuron [6], [35]. In contrast, simultaneously recorded VTA non-DA neurons (units 3 and 4) showed no significant responsiveness immediately after onset of the conditioned tone (Figure 6A).

Our results showed that VTA non-DA neurons exhibited clear rhythmic activity during wheel running (Figures 3, 4, 5). To determine whether the VTA DA neurons might play a role in wheel running, we did cross-correlation analyses of the simultaneously recorded putative DA and non-DA neurons (by using the most rhythmic non-DA neuron as the reference). As shown in Figure 6B, the cross-correlation analyses revealed that VTA putative DA neurons (units 1 and 2, the same two units as shown in Figure 6A) also exhibited rhythmic activity during wheel running, although to a lesser degree in compare with the non-DA neurons (units 3 and 4, the same two units as shown in Figure 6A). This is particularly interesting and further support the notion that activity of the VTA DA neurons plays a role in locomotor activity [31].

To determine whether the rhythmic activity of VTA putative DA neurons during wheel running was of significance, we compared the cross-correlation results between recorded spikes and shuffled spikes (randomized spikes). As shown in Figure 6C, there was no evident rhythmic activity of the shuffled DA units (the same unit 3 as shown in Figure 6B was used as the reference for cross-correlation analysis). Our correlation coefficient analyses (see Materials and Methods) revealed that there was significant difference between recorded spikes and shuffled spikes for both DA and non-DA neurons (*P*<0.001; Figure 6D), thus suggesting that both putative DA and non-DA neurons exhibited significant rhythmic activity during voluntary wheel running. Together, these findings suggest that both VTA putative DA and non-DA neurons are involved in processing motivational locomotor signals that correlate with animal's movement rhythms during wheel running.

### Burst activation of the VTA putative DA neuron at movement initiation/termination

**Figure 7:**
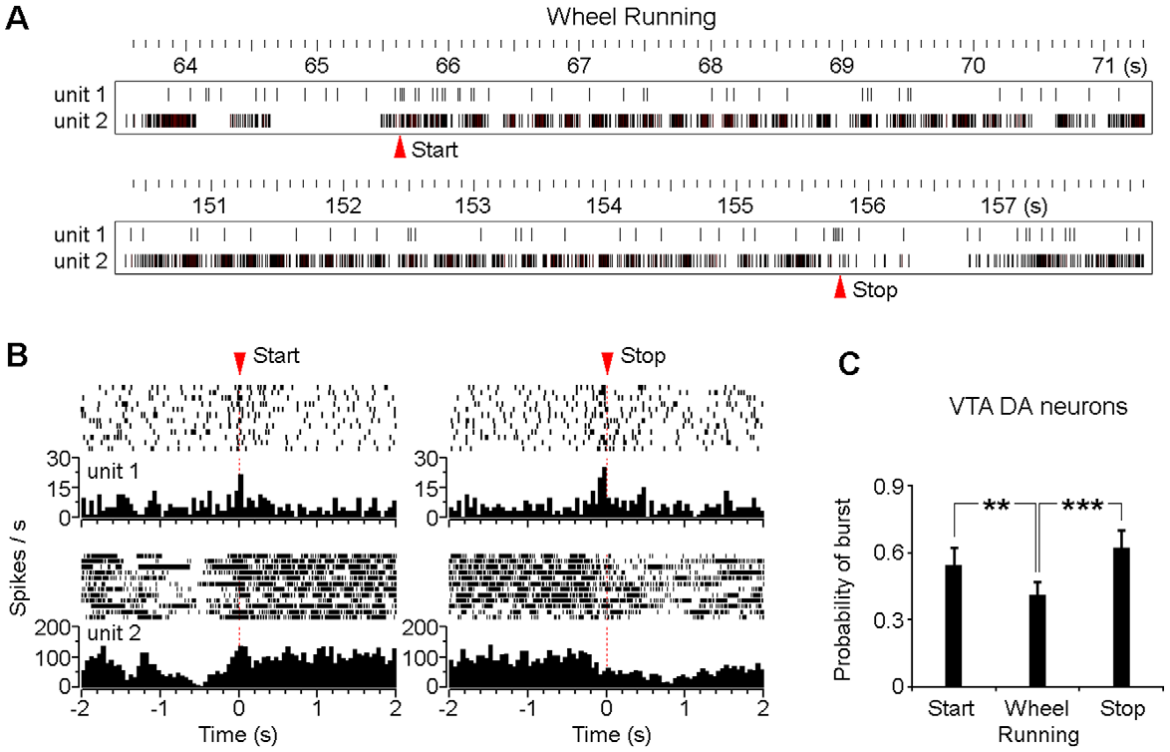
Burst activation of the VTA putative DA neuron at wheel running initiation/termination. (A) Rasters of two simultaneously recorded putative DA (unit 1) and non-DA (unit 2) neurons during voluntary wheel running. Red triangles indicate the start and stop of wheel running, respectively. It was noted that the two neurons were recorded from one tetrode in the VTA. (B) Peri-event rasters and histograms of the same two neurons (as shown in A) referenced to the starts (left panels) and stops (right panels) of wheel running. (C) VTA putative DA neurons exhibit significant higher probability of burst activity at starts and stops of wheel running in compare with the sustained wheel running. (n  =  14; **P < 0.01, ***P < 0.001, Student's paired *t*-test). Error bars represent s.e.m.

To further examine how the VTA neurons were related to motivational voluntary movement behaviors, we set out to analysis the neural activity at both the onset and offset of voluntary wheel running. As shown in Figure 7A, two units were recorded simultaneously from one tetrode in the VTA. The putative DA neuron (unit 1) exhibited significant burst activation at both the onset (start) and offset (stop) of wheel running, while the non-DA neuron (unit 2) showed significant rhythmic activity during wheel running (Figure 7A). Peri-event raters and histograms referenced to the start of wheel running indicated that there was significant burst activation of the putative DA neuron at the movement initiation (Figure 7B_left panels, unit 1). Similarly, the putative DA neuron exhibited significant burst activation at the termination of wheel running (Figure 7B_right panels, unit 1). Statistical analysis results suggest that VTA putative DA neurons showed significant higher probability of burst activity at both movement initiations and terminations in compare with the sustained wheel running (Figure 7C). This burst activation of the VTA DA neuron may provide motivational signals in generating start/stop signals for voluntary movements.

## Discussion

The above ensemble recordings and analyses have provided several novel insights into the role of VTA neuronal populations in processing both reward-related and locomotor-related motivational signals. First, VTA putative DA neurons exhibit significant burst activation in response to the presentation of reward predicting cues, as well as at voluntary movement initiations and terminations. This general response property of the DA neuron is consistent with the notion that dopamine activity is more associated with motivation or “wanting” rather than hedonic impact of reward [3], [10], [11]. The burst activation of the DA neuron in movement initiation/termination is particularly interesting since VTA DA neurons send majority of their outputs to downstream nucleus accumbens (NAcc), which is a key interface in translating motivation into action [36]. It will be of interest to further investigate the interaction between the VTA and NAcc neurons during motivated locomotor behaviors. Recently, it has been reported that neuronal activity in the nigrostriatal circuits emerged at the start/stop of sequence learning [37]. Together, DA neurons in both the VTA and SNc (substantia nigra pars compacta) seem to play an important role in generating start/stop signals during voluntary motor control. It is possible that VTA projection pathway is involved in motivational signaling while the SNc pathway is more associated with habit signaling [3].

Second, VTA putative DA neurons are involved in processing locomotor activity during sustained rhythmic movement such as wheel running. Although it is widely believed that the nigrostriatal DA pathway is important for animal's locomotor activity, the role for VTA DA neurons in locomotion remains equivocal [31]. Our analyses of simultaneously recorded VTA putative DA and non-DA neurons suggest that the activity of these putative DA neurons correlates well with animal's movement rhythms during wheel running (Figure 6). This locomotor-correlated activity may function in regulating or facilitating animal's movement behaviors or providing motivational signals in sustained rhythmic movement activities. Considering the role of dopamine transmitter in neural plasticity [38], these putative DA neurons are likely to play an important role in motor skill learning [34] and further mediating action-habit transformation [39], [40].

Third, VTA non-DA neurons play a significant role in processing locomotor-related information. Few attempts have been made to characterize the electrophysiology activity of the VTA non-DA neurons *in vivo* [21], [23], and the function of these non-DA neurons in reward circuitry and motivational behaviors is still not clear. This is partially due to the difficulty of characterization of non-DA neurons in the VTA because of the lack of a clear boundary between the VTA and surrounding nuclei. In our experiment, many non-DA neurons and putative DA neurons were recorded simultaneously from the same tetrodes (e.g., Figure 1B, Figure 7A), which strongly suggests that these non-DA neurons were likely to be located in the VTA area. It has been suggested that the activity of the VTA non-DA neuron is associated with the initiation of movements or onset of walking [21]. In agree with this notion, we find that VTA non-DA neurons show increased activity during movement compared with quiet wakefulness. Importantly, our wheel running experiment design allows us to be more quantitatively in examining the animal's locomotor activity such as the running cycle, and our results further suggest that VTA non-DA neurons are also involved in sustained locomotor activities that correlated with the animal's movement rhythms. Finally, our observations on the freely behaving animals suggest that VTA non-DA neurons may engage in a wide variety of movement-related behaviors and the functional correlates of these non-DA neurons during voluntary behaviors needs to be further more carefully examined.

In summary, we show that both VTA putative DA and non-DA neurons exhibit rhythmic activity that correlated with the animal's movement rhythm during voluntary behaving. Our multi-tetrode *in vivo* recording experiment provide strong evidence that VTA DA and non-DA neurons conjunctively engage in processing locomtor-related motivational signals that are associated with movement initiation, maintenance and termination.

## Materials and Methods

### Subjects

Male C57BL/6J mice (Jackson Laboratory) aged 3–6 months were used in this study. Full details of the study were approved by the Institutional Animal Care and Use Committee, Georgia Health Sciences University and covered under Protocol # BR07-11–001.

### Surgeries

A 32-channel (a bundle of 8 tetrodes), ultra-light (<1 g), movable (screw-driven) electrode array was constructed similar to that described previously [41]. Each tetrode consisted of four 13-µm diameter Fe-Ni-Cr wires (Stablohm 675, California Fine Wire; with impedances of typically 2–4 MÙ for each wire) or 17-µm diameter Platinum wires (90% Platinum 10% Iridium, California Fine Wire; with impedances of typically 1–2 MÙ for each wire). One week before surgery, mice (3–5 months old) were removed from the standard cage and housed in customized homecages (40×20×25 cm). On the day of surgery, mice were anesthetized with Ketamine/Xylazine (80/12 mg/kg, i.p.); the electrode array was then implanted toward the VTA in the right hemisphere (3.4 mm posterior to bregma, 0.5 mm lateral and 3.8–4.0 mm ventral to the brain surface) (Figure 1A) and secured with dental cement.

### Tetrode Recording and Units Isolation

Two or three days after surgery, electrodes were screened daily for neural activity. If no DA neurons were detected, the electrode array was advanced 40∼100 µm daily, until we could record from a putative DA neuron. Multi-channel extracellular recording was similar to that described previously [41]. In brief, spikes (filtered at 250–8000 Hz; digitized at 40 kHz) were recorded during the whole experimental process using the Plexon multichannel acquisition processor system (Plexon Inc. Dallas, TX). Mice behaviors were simultaneously recorded using the Plexon CinePlex tracking system. Recorded spikes were isolated using the Plexon OfflineSorter software: multiple spike sorting parameters (e.g., principle component analysis, energy analysis) were used for the best isolation of the tetrode-recorded spike waveforms.

### Reward Conditioning

Mice were trained to pair a tone (5 kHz, 1 sec) with subsequent food pellet delivery for at least two days in a reward chamber (45 cm in diameter, 40 cm in height). Mice received 40–60 trials for training per day, with an interval of 1–2 min between trials. The tone was generated by the A12–33 audio signal generator (5-ms shaped rise and fall; about 80 dB at the center of the chamber; Coulbourn Instruments). The sugar pellet (14 mg) was delivered by a food dispenser (ENV-203-14P, Med. Associates Inc.) and dropped into a sugar receptacle (12×7×3 cm) at the termination of the tone.

### Wheel Running

After recovery from surgery, mice were allowed access to a running wheel (13 cm in diameter; Figure 3A) for two or three days before experiment (10–30 min per day). Wheel-running was completely voluntary in our experiment (3–5 sessions per day; 10–30 min per session).

### Histological Verification of Recording Site

On completion of the experiments, the final electrode position was marked by passing a 10-sec, 20-µA current (Stimulus Isolator A365, WPI) through two tetrodes. Mice were deep anesthetized and perfused with 0.9% saline followed by 4% paraformaldehyde. Brains were then removed and post-fixed in paraformaldehyde for at least 24 h. Brains were rapidly frozen and sliced on a cryostat (50-µm coronal sections) and stained with cresyl violet. All the neurons used in the current analyses were estimated to be recorded from the VTA area (Figure 1A).

### Data Analysis

Sorted neural spikes were processed and analyzed in NeuroExplorer (Nex Technologies) and Matlab. DA neurons were classified based on the following criteria: 1) significant activation in response to the conditioned tone that predicted food pellet delivery; 2) low baseline firing rate (0.5–10 Hz) and long inter-spike interval (ISI >4 ms within a = 99.8% confidence level); 3) regular firing pattern when mice were freely behaving (fluctuation <3 Hz). Here, fluctuation represents the standard deviation (s.d.) of the firing rate histogram bar values (bin  = 1 sec; recorded for at least 600 sec). Neurons that did not meet these criteria were classified as non-DA neurons. It was noted that the majority of the classified putative DA neurons (9/10) tested showed significant suppression ( = 20% baseline firing rate), while the majority of the non-DA neurons (20/22) showed limited or no change of firing rate by the dopamine receptor agonist apomorphine (Figure 2C).

Neuronal activity changes during reward conditioning were compared against a 10-sec control period before onset of the conditioned tone in each trial with a constant time window (50–600 ms) after onset of the conditioned tone using a Wilcoxon signed-rank test. Auto-correlation, cross-correlation, power spectral density analyses were conducted in NeuroExplorer. Smoothing of histograms was conducted in NeuroExplorer using a Gaussian filter (filter width  = 5 bins). Shuffled neural spikes were generated in Matlab [42]. Start and stop of wheel running, limb-movement cycles during wheel running were scored offline by the experimenter from videotapes. Burst activity calculation of the DA neuron was similar to that described previously (burst onset, ISI of  = 80 ms; burst offset, ISI of  = 160 ms) [43]. Burst activity probabilities of the VTA DA neurons at starts and stops of wheel running were calculated in a time window of ±1 sec (Figure 7C).

For correlation coefficient analyses (Figure 6D), cross-correlation histograms were first normalized and z-scored. We then used a set of simulated sine oscillation curves y  =  sin (ax+b) as the reference for the calculation of correlation coefficient (conducted in Matlab using “corrcoef”). Here, a =  2*Π*/T; T was wheel running cycle. We screened the b values in each rhythm cycle to get the maximum correlation coefficient values for individual recorded and shuffled units. Significant differences were determined using student's paired *t*-test across unless noted otherwise.

## References

[1] Wise RA (2004). Dopamine, learning and motivation.. Nat Rev Neurosci.

[2] Schultz W (2007). Multiple dopamine functions at different time courses.. Annu Rev Neurosci.

[3] Ikemoto S, Panksepp J (1999). The role of nucleus accumbens dopamine in motivated behavior: a unifying interpretation with special reference to reward-seeking.. Brain Res Rev.

[4] Laviolette SR, Van Der Kooy D (2001). GABAA receptors in the ventral tegmental area control bidirectional reward signaling between dopaminergic and non-dopaminergic neural motivational systems.. Eur J Neurosci.

[5] Di Chiara G, Bassareo V (2007). Reward system and addiction: what dopamine does and doesn't do.. Curr Opin Pharmacol.

[6] Schultz W (2002). Getting formal with dopamine and reward.. Neuron.

[7] Bayer HM, Glimcher PW (2005). Midbrain dopamine neurons encode a quantitative reward prediction error signal.. Neuron.

[8] Pan WX, Schmidt R, Wickens JR, Hyland BI (2005). Dopamine cells respond to predicted events during classical conditioning: evidence for eligibility traces in the reward-learning network.. J Neurosci.

[9] Joshua M, Adler A, Mitelman R, Vaadia E, Bergman H (2008). Midbrain dopaminergic neurons and striatal cholinergic interneurons encode the difference between reward and aversive events at different epochs of probabilistic classical conditioning trials.. J Neurosci.

[10] Berridge KC, Robinson TE (1998). What is the role of dopamine in reward: hedonic impact, reward learning, or incentive salience?. Brain Res Rev.

[11] Cannon CM, Palmiter RD (2003). Reward without dopamine.. J Neurosci.

[12] Swanson LW (1982). The projections of the ventral tegmental area and adjacent regions: a combined fluorescent retrograde tracer and immunofluorescence study in the rat.. Brain Res Bull.

[13] Margolis EB, Lock H, Hjelmstad GO, Fields HL (2006). The ventral tegmental area revisited: Is there an electrophysiological marker for dopaminergic neurons?. J Physiol.

[14] Nair-Roberts RG, Chatelain-Badie SD, Benson E, White-Cooper HW, Bolam JP (2008). Stereological estimates of dopaminergic, GABAergic and glutamatergic neurons in the ventral tegmental area, substantia nigra and retrorubral field in the rat.. Neuroscience.

[15] Steffensen SC, Svingos AL, Pickel VM, Henriksen SJ (1998). Electrophysiological characterization of GABAergic neurons in the ventral tegmental area.. J Neurosci.

[16] Van Bockstaele EJ, Pickel VM (1995). GABA-containing neurons in the ventral tegmental area project to the nucleus accumbens in rat brain.. Brain Res.

[17] Carr DB, Sesack SR (2000). GABA-containing neurons in the rat ventral tegmental area project to the prefrontal cortex.. Synapse.

[18] Carr DB, Sesack SR (2000). Projections from the rat prefrontal cortex to the ventral tegmental area: target specificity in the synaptic associations with mesoaccumbens and mesocortical neurons.. J Neurosci.

[19] Fields HL, Hjelmstad GO, Margolis EB, Nicola SM (2007). Ventral tegmental area neurons in learned appetitive behavior and positive reinforcement.. Annu Rev Neurosci.

[20] Steffensen SC, Lee RS, Stobbs SH, Henriksen SJ (2001). Responses of ventral tegmental area GABA neurons to brain stimulation reward.. Brain Res.

[21] Lee RS, Steffensen SC, Henriksen SJ (2001). Discharge profiles of ventral tegmental area GABA neurons during movement, anesthesia, and the sleep-wake cycle.. J Neurosci.

[22] Stobbs SH, Ohran AJ, Lassen MB, Allison DW, Brown JE (2004). Ethanol suppression of ventral tegmental area GABA neuron electrical transmission involves N-methyl-D-aspartate receptors.. J Pharmacol Exp Ther.

[23] Miller JD, Farber J, Gatz P, Roffwarg H, German DC (1983). Activity of mesencephalic dopamine and non-dopamine neurons across stages of sleep and walking in the rat.. Brain Res.

[24] Puryear CB, Kim MJ, Mizumori SJ (2010). Conjunctive encoding of movement and reward by ventral tegmental area neurons in the freely navigating rodent.. Behav Neurosci.

[25] Sherwin CM (1998). Voluntary wheel running: a review and novel interpretation.. Anim Behav.

[26] Rhodes JS, Gammie SC, Garland T (2005). Neurobiology of mice selected for high voluntary wheel-running activity.. Integr Comp Biol.

[27] Belke TW, Wagner (2005). The reinforcing property and the rewarding aftereffect of wheel running in rats: a combination of two paradigms.. Behav Proc.

[28] Hyland BI, Reynolds JN, Hay J, Perk CG, Miller R (2002). Firing modes of midbrain dopamine cells in the freely moving rat.. Neuroscience.

[29] Robinson S, Smith DM, Mizumori SJ, Palmiter RD (2004). Firing properties of dopamine neurons in freely moving dopamine-deficient mice: effects of dopamine receptor activation and anesthesia.. Proc Natl Acad Sci USA.

[30] Roesch MR, Calu DJ, Schoenbaum G (2007). Dopamine neurons encode the better option in rats deciding between differently delayed or sized rewards.. Nat Neurosci.

[31] Beninger RJ (1983). The role of dopamine in locomotor activity and learning.. Brain Res Rev.

[32] Kalivas PW, Stewart J (1991). Dopamine transmission in the initiation and expression of drug- and stress-induced sensitization of motor activity.. Brain Res Rev.

[33] Flöel A, Breitenstein C, Hummel F, Celnik P, Gingert C Dopaminergic influences on formation of a motor memory.. Ann Neurol.

[34] Molina-Luna K, Pekanovic A, Röhrich S, Hertler B, Schubring-Giese M (2009). Dopamine in motor cortex is necessary for skill learning and synaptic plasticity.. PLoS One.

[35] Redgrave P, Gurney K, Reynolds J (2008). What is reinforced by phasic dopamine signals?. Brain Res Rev.

[36] Mogenson GJ, Jones DL, Yim CY (1980). From motivation to action: functional interface between the limbic system and the motor system.. Prog Neurobiol.

[37] Jin X, Costa RM (2010). Start/stop signals emerge in nigrostriatal circuits during sequence learning.. Nature.

[38] Jay TM (2003). Dopamine: a potential substrate for synaptic plasticity and memory mechanisms.. Prog Neurobiol.

[39] Everitt BJ, Robbins TW (2005). Neural systems of reinforcement for drug addiction: from actions to habits to compulsion.. Nat Neurosci.

[40] Wickens JR, Horvitz JC, Costa RM, Killcross S (2007). Dopaminergic mechanisms in actions and habits.. J Neurosci.

[41] Lin L, Chen G, Xie K, Zaia KA, Zhang S (2006). Large-scale neural ensemble recording in the brains of freely behaving mice.. J Neurosci Methods.

[42] Narayanan NS, Laubach M (2009). Methods for studying functional interactions among neuronal populations.. Methods Mol Biol.

[43] Grace AA, Bunney BS (1984). The control of firing pattern in nigral dopamine neurons: burst firing.. J Neurosci.

[44] Paxinos G, Franklin KBJ (2001). The mouse brain in stereotaxic coordinates, ed. 2..

